# Policy Versus Practice: Facilitators and Barriers of Chronic Care Integration in Dutch General Practice – a Survey Study

**DOI:** 10.5334/ijic.8443

**Published:** 2024-12-18

**Authors:** Toine E. P. Remers, Simone A. Van Dulmen, Erik W. M. A. Bischoff, Florien M. Kruse, Marcel G. M. Olde Rikkert, Patrick P. T. Jeurissen

**Affiliations:** 1 Radboud university medical center, Department of IQ Health, Nijmegen, The Netherlands; 2 Radboud university medical center, Department of Primary and Community Care, Nijmegen, The Netherlands; 3 Dutch Ministry of Health, Welfare, and Sport, Department Macro-Economic Affairs and Labour Market, the Hague, The Netherlands; 4 Radboud university medical center, Department of Geriatric Medicine, Nijmegen, The Netherlands; 5 Radboud university medical center, Donders Institute for Brain Cognition and Behaviour, Department of Geriatric Medicine, Radboud Alzheimer Centre, Nijmegen, The Netherlands

**Keywords:** multimorbidity, integrated care, primary care, care groups, health policy, general practitioners

## Abstract

**Introduction:**

Multimorbidity challenges quality and sustainability of healthcare systems. Care groups were introduced in the Netherlands to promote integration of chronic primary care, but it remains unknown to which degree they facilitate this. This study therefore aims to determine whether Dutch general practices perceive themselves to be capable of delivering integrated chronic care and uncover the role of care groups.

**Methods:**

We performed a survey study amongst 39 care groups and 65 healthcare providers within general practices (GPs and nurse practitioners).

**Results:**

43% of healthcare providers within general practices are (very) dissatisfied with capabilities for chronic care to patients and 56% do not feel capable of delivering integrated care. Care groups and providers show alignment in their perception of some of the most important facilitators and barriers such as motivation and lack of time, but other factors are valued differently at both levels.

**Discussion:**

Our findings show inability of general practices to deliver integrated chronic care despite a health system that is inherently supportive of care integration and point to a mismatch between barriers and facilitators amongst practices and care groups, resulting in providers partly relying on their motivation in accommodating integrated chronic care.

**Conclusion:**

General practices are not sufficiently supported by care groups and national policies in delivering integrated chronic care. The identified mismatch between policy and practice warrants redesign of support from care groups to align policies with identified barriers and facilitators at the provider level.

## Introduction

The rising incidence of people with multimorbidity is recognised as a prominent challenge healthcare will face in the coming decades [1,2,3]. The simultaneous presence of multiple chronic conditions within a single individual does not align with the current, still highly fragmented healthcare systems that are typically organised around specific diseases [1,2,4]. This results in poor coordination of individual patient care, which can lead to unnecessary and potentially harmful care due to polypharmacy, conflicting treatments, or the overlooking of symptoms that are not necessarily related to one chronic condition but arise from interactions between these diseases [1,2,5]. This not only leads to lower quality of care but also contributes to rising healthcare costs for this patient group [6], which is already responsible for a large share of total healthcare expenditures [6,7]. Health systems worldwide therefore need to identify opportunities to deliver a higher degree of integrated care to these patient groups. In this instance, integrated care is seen as patient care that involves optimal coordination between involved healthcare providers through effective and efficient communication, coordination, and cooperation. Such integration ultimately serves to improve the continuity and quality of care for the patient, and cooperation also takes place across different sectors [8,9].

General practices are seen as having a crucial role and vital experience in integrating the fragmented care patterns of this patient group due to their generalist approach to patients [1,10,11]. As gatekeeper in many healthcare systems, patients often have frequent contact with their general practitioner (GP), making it possible to detect early signs of failing coordination whilst serving as the care integrator for this patient group [10]. When patients are well-managed by general practices, significant healthcare costs can be saved in other sectors as well [12]. Although general practitioners are considered very successful in their role as gatekeepers within the healthcare systems that have assigned them this role [13], GPs have also been shown to face challenges in conducting integration due to short consultation times, payment per consultation, and disease-oriented treatment guidelines [2,10,11,14].

Traditionally, the setup of the Dutch primary healthcare sector has been supportive of care integration, and this long-standing commitment has fostered an environment conducive to innovative care models and collaborative approaches to patient management [15]. General practices in the Netherlands were mandated to merge into overarching care groups from 2007 onwards to strengthen care integration and promote uniform care for patients with chronic conditions across practices, arrange after-hours care, and support care innovations within primary care [16,17]. These care groups were introduced as additional governance level that enabled overarching assistance of individual general practices with their increasingly complex tasks. Besides arranging after-hours care, care groups bear financial and clinical responsibility for patients with chronic conditions enrolled in their underlying practices and can freely negotiate with healthcare insurers about prices for services related to these patient groups [16,17]. Care groups receive a substantial share of the annual national primary care budget of more than 4 billion euros based on these negotiations (>10% of total budget) [18,19]. In turn, care groups can employ a wide range of innovations and activities amongst their general practices to e.g., promote integrated care. Among other things, these reforms led to the nationwide introduction of bundled payments within care groups for the delivery of a predefined set of services to patients with diabetes, COPD, or cardiovascular risk management in 2010 [20,21,22]. Early results of these payment bundles were promising and showed improvements in coordination of care and stricter adherence to treatment protocols as intended [21]. However, a long-term evaluation showed that these bundles actually increased healthcare costs; especially for patients with multimorbidity [23]. Also, GPs previously stated to consider after-hours care run by care groups as a substantial source of unnecessary care, while the restructuring into care groups was intended to do exactly the opposite [24]. Although the policy framework in Dutch primary care is considered supportive and promising, integration of care for patients with multimorbidity by general practices through support from care groups therefore still seems to remain suboptimal in practice in the Netherlands.

To improve this, national agencies have recently published several documents targeted at directions towards integrated care for patients with multimorbidity in Dutch general practices [8,25]. However, a recent qualitative study within one care group showcased that well-known difficulties such as lack of time and disease-oriented payments remained [26]. This points to continued difficulties for general practices in providing integrated care to patients with multimorbidity in daily practice and a lack of knowledge of whether care groups are currently sufficiently facilitating this. To further uncover these difficulties and study perceived capability at the provider level, the goal of this study is to describe the extent to which healthcare providers within Dutch general practices perceive themselves capable of delivering integrated chronic care to patients with multimorbidity in daily practice and the experienced barriers and facilitating factors related to this. Furthermore, we aim to provide insights into the perceived contributions from care groups regarding care integration and specific actions that could be required within this area.

## Methods

### Study design

This study applied an observational, cross-sectional study design with data collection at two time points through an online questionnaire sent to care groups and individual care professionals within general practices. Textbox 1 shows an operationalisation of the broad concepts of integrated care and patients with multimorbidity that were applied throughout both questionnaires [1,8,9,27].

**Textbox 1 Operationalisation of integrated care and multimorbidity that were applied throughout both questionnaires, including references to literature on which operationalisations were based.**

- Integrated care: patient care that involves optimal coordination between involved healthcare providers through effective and efficient communication, coordination, and cooperation. Integration ultimately serves to improve the continuity and quality of care for the patient. Cooperation also takes place across different sectors [8,9].
- Multimorbidity: having two or more chronic conditions [1,27]. However, integrated chronic care may also benefit patients with one chronic condition when standards of care are not sufficient because of multiple parallel care pathways and/or coexisting complex psychosocial conditions [1,26]. Therefore, in our questionnaire, we operationalised our target population as: “patients with multimorbidity or patients with one chronic condition and complex care needs”.

### Study population and data collection

Questionnaires were targeted at representatives of care groups and healthcare providers within general practices. For care groups, any (program) manager who was most concerned with innovative care programs for chronic conditions was eligible for inclusion. Care groups were given the discretionary power to determine whoever in their organisation was most suited for this. For general practices, any healthcare professional working in a general practice (i.e., GP, nurse practitioner, physician assistant) was eligible for inclusion.

As far as our knowledge goes, there are no validated questionnaires available for assessing (readiness for) delivery of integrated care to patients with multimorbidity in general practices, meaning we had to develop the questionnaires ourselves. We developed online questionnaires in LimeSurvey through discussions with experts on relevant themes and by consulting literature on barriers to delivery of integrated care to patients with multimorbidity in general practice [11,28,29]. Insights from these consultation processes were translated into a list of relevant barriers and facilitators related to delivery of integrated, additional relevant closed-ended statements, and open-ended questions for broad themes of care such as opinions on the current situation or future needs. Both questionnaires were subsequently piloted amongst peers to ensure face validity, meaning the questions were valid and applied to our groups of respondents, the right terminology was used, and lists of e.g. barriers and facilitators were comprehensive and applicable [30,31]. Three randomly selected care groups and two GPs known to the research team were included in this pilot phase. Adjustments were made based on feedback from these participants through 30-minute online meetings. Adjustments mostly concerned different wording for specific terminology, refinements of explicit definitions for terms such as “integrated care”, and inclusion of additional barriers and facilitators.

Eventually, questionnaires contained 20 and 17 questions across four domains related to integrated care delivery for care groups and general practices, respectively (see supplementary file 1). A five-point Likert scale was applied for close-ended statements [32] with optional open fields in case participants wanted to provide additional information. Open-ended questions were added for domains that specifically aimed to generate new insights, views, or suggested directions of participants.

The questionnaires were distributed to representatives of 64 care groups at once in March 2023 through the national association of care groups InEen. Subsequently, healthcare providers within general practices were approached in May and June 2023 via direct requests from care groups that expressed their willingness to cooperate in the distribution after completing their own questionnaire. To generate additional responses, the questionnaire for general practices was also disseminated on social media. Both groups of respondents were given six weeks to complete the questionnaire after the initial invitation was sent. Repeated participation was prevented through personalised links for care groups and descriptive characteristics for general practices. Both care groups and healthcare providers within general practices gave written informed consent prior to participation which explained that data would be anonymised and analysed at group level. Confidential and anonymised data was subsequently stored separately following Dutch regulations concerning general data protection. This research was performed in accordance with the Declaration of Helsinki and was determined to be in accordance with the applicable legislation concerning reviewal by an accredited research ethics committee such as Medical Research involving Human Subjects Act and Medical Treatment Contracts Act (ref. 2024-17620).

### Data analysis

Survey data was transported to Microsoft Excel and summarised per answer category for closed-ended statements. Answers to open-ended questions were categorised by theme using Excel, and overarching themes related to specific topics were identified through a basic content analysis of these categorised answers. Answers of partially completed questionnaires were included up to the last fully completed domain. For barriers and facilitators, each participant’s top five answers were weighted to give an accurate representation of the stated relative importance of all factors across participants. The number one barrier and facilitator were assigned five points up to the number five answer being assigned one point, and so on. Weighted percentages of barriers and facilitators were subsequently calculated by dividing the total number of points assigned per barrier/facilitator by the total sum score of points across all barriers or facilitators. This enabled the relative importance of factors to be included in our results over simple visualisations of rankings per factor. In doing so, a barrier/facilitator that is ranked as second or third most important by a lot of respondents will have a higher weighted rank than a barrier that is ranked first in some instances and not ranked for most other participants.

## Results

A response was received from 39 out of the 64 care groups eligible for inclusion (61% response rate). 44% (n = 17) of the care group respondents were (program) managers, 10% (n = 4) were bundled care coordinators, 8% (n = 3) were medical directors, and 38% (n = 15) indicated that they had a position that fell outside the options on the questionnaire, with the position of chronic care team leader being mentioned most often. On average, care groups represented 74 general practices (range 1–260). Within general practices, 72 healthcare providers started participation, of which 65 fully completed the questionnaire (response rate unknown). In line with national averages [33], a majority of respondents within general practices concerned GPs and 72% were female (see Table 1). Stated reasons for not completing participation were the length of the questionnaire (for general practices) or not having a strategic focus on the patient group (for care groups).

**Table 1:** Characteristics of the respondents of the general practice questionnaire.


CHARACTERISTIC	N (%)

**Gender**	

*Male*	20 (28%)

*Female*	52 (72%)

**Function**	

*General practitioner*	53 (74%)

*Somatic nurse practitioner*	16 (22%)

*Mental health nurse practitioner*	0 (0%)

*Other*	3 (4%)

**Working experience**	

*0–5 years*	5 (7%)

*5–10 years*	9 (13%)

*10–15 years*	12 (17%)

*15–20 years*	18 (26%)

*More than 20 years*	26 (37%)


Table 2 shows answers within both questionnaires on perceived ability to deliver (integrated) care or the degree to which bundled care programs amongst care groups might promote this. 43% (n = 31) of the surveyed healthcare providers within general practices were (very) dissatisfied with the current capabilities within the Dutch system to deliver care to patients with multimorbidity and 56% (n = 38) did not agree with the statement that they felt capable of delivering integrated care to this patient group. At the care group level, 53% (n = 21) of the respondents (strongly) agreed with the statement that the implementation of bundled payments for specific conditions in the Dutch healthcare system in 2010 encourages integrated care. Also, 66% (n = 25) (strongly) agreed with the statement that these bundled payments sufficed for the delivery of care to patients with chronic conditions.

**Table 2:** Answers to four questions within both questionnaires that specifically focussed on the ability to deliver (integrated) care and the degree to which bundled care programs amongst care groups might have promoted this.


QUESTION (WITHIN QUESTIONNAIRE FOR GENERAL PRACTICES/CARE GROUPS)	N (PERCENTAGE OF TOTAL)

**How satisfied are you with the ability of the Dutch healthcare system to provide care to patients with complex health problems (general practices)?**	**72 (100%)**

*Very satisfied*	2 (3%)

*Satisfied*	18 (25%)

*Neutral*	21 (29%)

*Dissatisfied*	24 (33%)

*Very dissatisfied*	7 (10%)

**I feel capable of delivering integrated care to patients with complex care patterns (general practices)**	**67 (100%)**

*Strongly agree*	3 (4%)

*Agree*	26 (39%)

*Not agree/nor disagree*	26 (39%)

*Disagree*	11 (16%)

*Strongly disagree*	1 (1%)

**The national implementation of the general concept of bundled payments for specific conditions encourages integrated care for patients with multimorbidity**	**38 (100%)**

*Strongly agree*	8 (21%)

*Agree*	12 (32%)

*Not agree/nor disagree*	5 (13%)

*Disagree*	11 (29%)

*Strongly disagree*	2 (5%)

**The general concept of bundled payments for specific conditions is sufficient for providing care to patients with chronic conditions – in terms of content (care groups)**	**38 (100%)**

*Strongly agree*	6 (16%)

*Agree*	19 (50%)

*Not agree/nor disagree*	7 (18%)

*Disagree*	4 (11%)

*Strongly disagree*	2 (5%)


**Figure 1:**
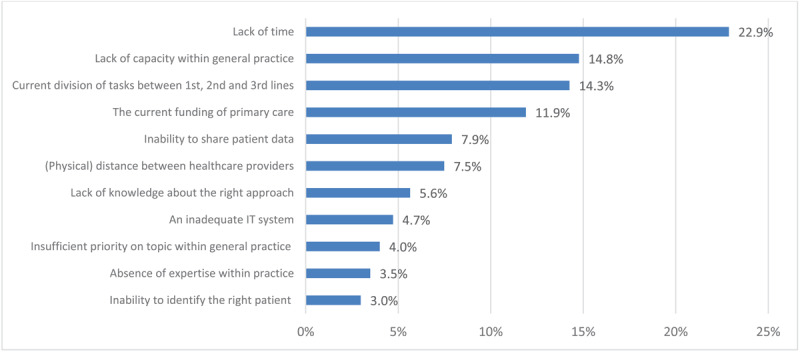
A weighted ranking of barriers in providing integrated care to patients with multimorbidity, as experienced by healthcare providers within general practices. Weighted percentages of barriers were calculated by assigning points (5 until 1) to barriers according to their ranking amongst an individual’s top five and subsequently dividing the total number of points assigned per barrier by total sum score of points across all barriers.

**Figure 2:**
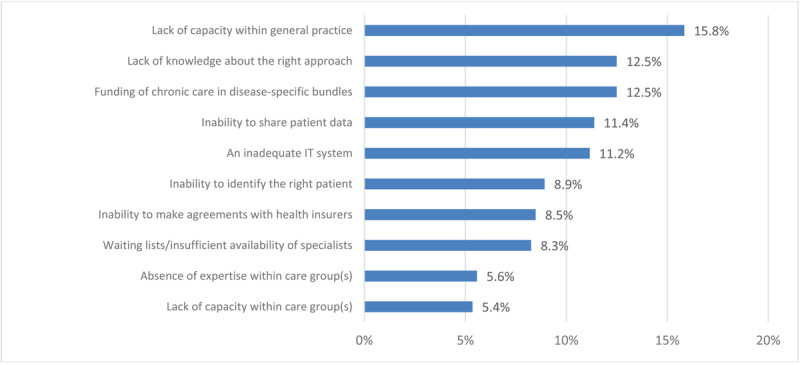
A weighted ranking of barriers in providing integrated care to patients with multimorbidity, as experienced by care groups. Weighted percentages of barriers were calculated by assigning points (5 until 1) to barriers according to their ranking amongst an individual’s top five and subsequently dividing the total number of points assigned per barrier by total sum score of points across all barriers.

Figures 1 and 2 show the weighted ranking of barriers experienced by healthcare providers within general practices and care groups regarding the delivery of integrated care. Lack of time (22.9%) and capacity (14,8%), current division of tasks between primary care and other sectors (14,3%), and the current funding of primary care (11,9%) were the top four highest-ranking barriers among healthcare providers within general practices. Care groups mentioned capacity within general practices (15,8%), lack of knowledge about the right approach (12,5%), current financing through disease-specific bundles (12,5%), and inability to share patient data (11,2%) as top four most important barriers. Both figures also show distinct differences in valuations of barriers between both participant groups. Care groups deemed lack of knowledge about the right approach, an inadequate IT system, and inability to identify the right patient as fairly influential barriers whilst valuations of healthcare providers within general practices placed most of those barriers amongst the least influential of all.

**Figure 3:**
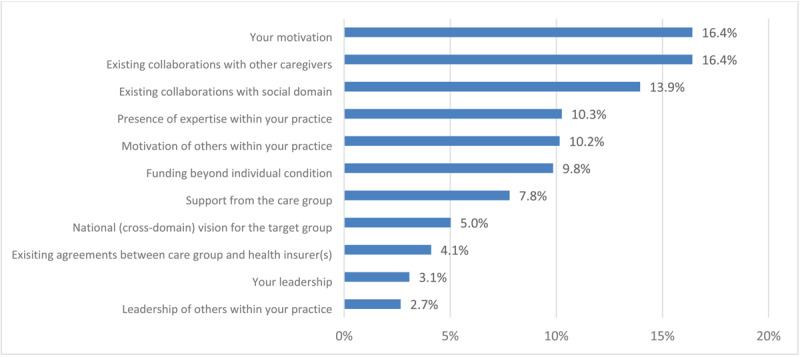
A weighted ranking of facilitating factors in providing integrated care to patients with multimorbidity, as experienced by healthcare providers within general practices. Weighted percentages of facilitators were calculated by assigning points (5 until 1) to facilitators according to their ranking amongst an individual’s top five and subsequently dividing the total number of points assigned per facilitator by total sum score of points across all facilitators.

**Figure 4:**
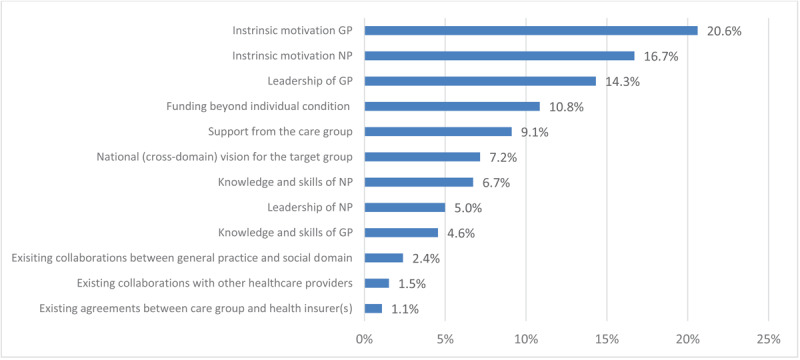
A weighted ranking of facilitating factors in providing integrated care to patients with multimorbidity, as experienced by care groups. Weighted percentages of facilitators were calculated by assigning points (5 until 1) to facilitators according to their ranking amongst an individual’s top five and subsequently dividing the total number of points assigned per facilitator by total sum score of points across all facilitators. Abbreviations: GP: general practitioner; NP: nurse practitioner.

Figures 3 and 4 show the weighted ranking of facilitators experienced by healthcare providers within general practices and care groups regarding delivery of integrated care. Own motivation (16,4%), existing collaborations with other caregivers (16,4%), and collaborations with the social domain (13,9%) were the top three highest-ranking facilitators for delivery of integrated care amongst healthcare providers within general practices. Care groups mentioned motivation of GPs (20,6%) and NPs (16,7%) and leadership of GPs (14,3%) as the top three most important facilitators. Both figures also show large differences in valuations of facilitators amongst both participant groups. Care groups deem leadership of GPs very important and state the value of existing collaborations as least important whilst healthcare providers within general practices state the exact opposite for these two facilitators.

Basic content analysis of categorised open-ended questions revealed that 49% of the responding care groups had programs to promote integrated care for patients with multimorbidity. The scope and degree of support from such programs largely varied. Some care groups merely provided periodic educational activities amongst their general practices whilst others employed comprehensive strategies focussing on several aspects simultaneously, including agreements with healthcare insurers, specific care plans for this patient group, and cross-sector collaborations. Healthcare providers within general practices mostly indicated a need for national guidance and stewardship in realising appropriate financing for delivery of care to this patient group, enabling cross-sector collaborations, and major shifts in the organisation of healthcare systems and divisions of tasks.

## Discussion

### Summary of findings

Our study shows that a majority of surveyed individual care professionals in Dutch general practices are dissatisfied with the current system’s ability to offer optimal care to patients with multimorbidity. 43% of these healthcare providers are (very) dissatisfied with the current capabilities to deliver care to patients with multimorbidity and 56% do not feel capable of delivering integrated care to this patient group. At the same time, a majority of care group respondents feel that bundled payments for specific conditions as implemented in the Dutch healthcare system in 2010 promote care integration and suffice for delivery of care to patients with chronic conditions. Similar barriers to delivery of integrated care are expressed amongst general practices and care groups, but there are also distinct differences. Common barriers include time and capacity constraints in general practices, current funding for primary care, and limitations in data sharing. A lack of knowledge is perceived as one of the major barriers amongst care groups, while it is one of the least mentioned barriers within general practices. Motivation of individual healthcare providers and appropriate funding are considered important promoting factors for delivery of integrated care amongst both groups, but there are distinct variations in the value placed on leadership and existing collaborations with other healthcare providers and the social sector in the delivery of integrated care. Programs to facilitate integrated care initiated by care groups mainly align with the mentioned barriers and promoting factors amongst those care groups whilst healthcare providers within general practices mostly express the need for system-level changes in organisation and financing of healthcare.

### Implications

Several reviews have pooled findings of qualitative studies to identify barriers of GPs concerning the management of patients with multimorbidity [10,14]. These studies primarily indicate to the current limitations of disease-oriented systems and guidelines that do not align with multimorbidity as found in our study as well. Despite not receiving adequate support at a system level, GPs were found to still be committed to building long-term relationships with patients with multimorbidity as they felt this could promote trust and was likely to provide deeper insights into the unique circumstances of a patient [10]. These findings appear consistent with the findings of this study, wherein general practices and care groups highlight motivation of healthcare providers within general practices as key facilitators for the establishment of integrated care. This indicates that general practices currently perceive limited support and largely rely on their motivation to deliver some degree of integrated care, nonetheless. However, besides finding barriers and mechanisms similar to previous studies, our study is the first to identify differences in experienced barriers amongst general practices and care groups that could indicate to a partial mismatch between these two organisational levels. For example, whilst general practices deem collaborations with others very useful, care groups consider those amongst the least facilitating factors for integrated care. This study also shows that almost 50% of care groups do not offer specific support to promote care integration for patients with multimorbidity among general practices. Care groups should therefore directly apply the results from this study in shaping targeted interventions that support delivery of integrated care amongst their general practices that truly align with barriers and facilitators amongst those general practices. In doing so, care groups should make use of their unique position in the Dutch provider landscape, which enables them to negotiate with insurers about the allocation of substantial resources for such supporting actions [16]. Recent findings regarding the possibilities of transforming disease-specific payment bundles into person-centred payments can provide important additional knowledge in reshaping such supportive actions [34].

With regards to existing evidence on the role of Dutch care groups in establishing integrated care for patients with multimorbidity, an earlier study amongst a Dutch care group demonstrated that several barriers to care integration remained despite specific programmatic support targeted at person-centred care delivery for patients with multimorbidity [26]. Barriers are mostly related to legislation, inadequate IT systems, and financial constraints [26]. Although these findings seem in line with our study, this qualitative study based its results on a small number of interviews with caregivers within a single care group that was actively enrolling a program to support personalised care among their general practices [26]. In contrast, our study included a variety of healthcare providers amongst general practices from different care groups and surveyed a wide range of care groups that showed considerable variation in the level of support provided. This enables us to provide a more balanced overview of barriers and facilitators experienced amongst general practices that might experience different degrees of support from their care groups. Nevertheless, our findings seem to confirm the earlier findings that barriers to integrated chronic care amongst general practices remain despite programmatic support and the Dutch primary care policy framework that is generally seen as highly facilitating for professional and organisational integration already [15]. However, more importantly, our study also demonstrates a distinct difference in valuation of these barriers and facilitators between general practices and care groups. Our findings therefore particularly highlight the importance of involving general practices in shaping actions within care groups or even at the national level. Recent efforts around the development of person-centred payment bundles within Dutch care groups that explicitly involved patients and healthcare providers within general practices provide an example of such a strategic approach that has been jointly developed [35].

### Strengths and limitations

A strength of this study is the considerable number of care groups that were surveyed and the different types of caregivers within general practices that were included from several care groups. This provides a more balanced perspective compared to focusing on a single care group or one specific type of caregiver within general practices, for example [26]. Also, including both care groups and healthcare providers within general practices enabled us to show novel insights into different perceptions of barriers and facilitators for integration of care and the limited role care groups currently seem to have in promoting integrated care. Lastly, by asking for rankings, respondents are forced to prioritise. This creates an overview of the relative importance of a wide range of pre-established factors rather than a few factors that may have been mentioned by respondents themselves.

This study also has some limitations. Most notably, there were few responses from healthcare providers within general practices, possibly due to the approach via care groups. Also, care groups and healthcare providers within general practices probably look at concepts such as integrated care through different lenses (administrator/manager v.s. clinical) This makes generalising the results to a broader context challenging. However, identified barriers are consistent with previous studies in the Dutch and international settings and responses are collected across multiple care groups, indicating that at least one context was not solely influential. This is also reflected by the characteristics of healthcare providers within general practices that seem to be in line with overall characteristics of GPs in terms of gender and age [33]. The results of this study, especially the differences in opinions and beliefs amongst general practices and care groups that might stem from looking at concepts through different lenses, can therefore still provide valuable insights for a broader context and determine directions for future research and policy.

Another limitation could be the fact that the use of a self-developed questionnaire with many closed-ended questions did not result in an in-depth understanding of certain concepts or insights into how certain factors influence the delivery of integrated care. However, the research’s goal was to obtain a general overview of perceived barriers and factors at two levels of primary care, which enables us to draw conclusions on the alignment of policy to promote integrated care at the care group level and the way such policies translated to actual practice within general practices. Additionally, open-ended questions were also added to topics where more extensive responses could provide additional insights.

### Conclusions

There has been a significant emphasis on general practices for streamlining of care to patients with multimorbidity to increase their quality of care and lower their significant contribution to overall healthcare costs in healthcare systems worldwide. Yet, multiple international studies have shown that general practices remain to receive little support in living up to that task over the past decades, even in the Dutch primary care system which is considered strongly supportive of professional and organisational integration. Results from this study confirm this and provide insights into the limited ability to deliver such integrated care as experienced by general practices in the current healthcare landscape. Our study also presents a deeper exploration of barriers and facilitators amongst general practices and care groups related to this integration of care and shows distinct differences in the valuation of certain aspects between these two policy levels. The insights from this study legitimise detailed assessments from care groups amongst their general practices into the prerequisites and needs for suitable support in facilitating integration of care for patients with multimorbidity. Specific areas of focus for care groups based on findings amongst general practices include freeing up time and capacity within practices, stimulating collaborations with other caregivers, and shaping adequate financial compensation for integrated care efforts. On a national level, our study highlights the current mismatch between existing policies and the desired integration in practice, emphasising the need for substantial reconsiderations of the current financing structure of general practices and the role care groups currently play in this. Altogether, the findings in our study should lead to a significant reconsideration and redesign of the support general practices are currently receiving from both care groups and national institutions to promote integration of care for patients with multimorbidity.

## Additional File

The additional file for this article can be found as follows:

10.5334/ijic.8443.s1 Supplementary File 1. Questionnaire for general practices and care groups.
