# Supplementary material for: Policy Versus Practice: Facilitators and Barriers of Chronic Care Integration in Dutch General Practice – a Survey Study
Source: Int J Integr Care. 2024 Dec 18;24(4):13. doi: 10.5334/ijic.8443 (PMC11661053; doi:10.5334/ijic.8443)
Supplement: Supplementary File 1. — Questionnaire for general practices and care groups. [file ijic-24-4-8443-s1.pdf]

Supplementary file 1: Questionnaire for general practices and care groups

General practice questionnaire

[Consent Statement]

"Please read the information below and agree to participate in this study before proceeding.

This questionnaire and your responses will be used for IQ healthcare scientific research purposes. Answers will be analysed and published anonymously and at a group level. Therefore, your answers will never be traceable to you as an individual or practice. Responses to this questionnaire will be stored for 15 years in accordance with Dutch laws and regulations governing scientific research. Results of this research will be processed in a scientific publication that is publicly accessible. Please give your consent to participate below.

Please note: Without consent, this research cannot be conducted and the questionnaire will be completed immediately."

**Domain 1: General practice characteristics**	
Question 1: What are the 4 digits of your general practice's zip code?	Fill-in question
Question 2: What is your position within the practice?	General practitioner/nurse practitioner-somatic/nurse practitioner-mental health/other (describe below)
Question 3: How many years have you been working within this position?	0-5 years/5-10 years/10-15 years/15-20 years/20+ years
Question 4: What is your gender?	Male/Female/Other
Question 5: Approximately how many patients are registered at your general practice?	… patients
Question 6: How many employees by specific position are employed within your practice?	General practitioner:<br>Nurse practitioner-somatic:<br>Nurse practitioner-mental health:<br>Physician assistant:<br>Support worker:<br>Other:

**Domain 2: Patients with complex health problems.**<br>This questionnaire focuses specifically on patients with complex health problems. By complex health problems we mean: having one or more chronic conditions where (general practitioner) care standards do not suffice due to multiple parallel care pathways and/or complex psychosocial conditions.<br><br>*Note: this does not only have to concern a patient with multimorbidity. It involves all patients with chronic conditions whose pattern of care you would describe as complex and for whom you feel that care according to standards is inadequate. So, for example, a patient with only diabetes but with challenging psychosocial conditions that requires you to provide care outside of standards may also fall under this definition.*	
Question 7: Statement: I agree with the definition of a patient with complex health problems as stated above.<br><br>(If agreeing to strongly disagree on question 8, make question 9 appear)	Completely agree - agree - neither agree nor disagree - disagree - completely disagree
Question 8: Please describe any modifications you may have to the above definition of a patient with complex health problems.	Open question
Question 9: What percentage of all patients within your practice falls under the above definition? This may be a rough estimate.	**… %**

Question 10*: What percentage of the patients you speak to as an individual provider in an average week fall under the above definition? This may be a rough estimate.	**… %**
Question 11: How satisfied are you with the ability of the Dutch healthcare system to provide care to patients with complex health problems?	Very satisfied, satisfied, neutral, dissatisfied, very dissatisfied

**Domain 3: Integrated care**<br>*Integrated care:* patient care that involves optimal coordination between involved healthcare providers through effective and efficient communication, coordination, and cooperation. Integration ultimately serves to improve the continuity and quality of care for the patient. Cooperation also takes place across different sectors.	
Question 12: Statement: I feel able to provide integrated care to patients with complex health problems.	Completely agree - agree - neither agree nor disagree - disagree - completely disagree
Question 13: The list below includes factors mentioned in literature and previous research as impeding integrated care. Make a top 5 from these factors that you experience in daily practice as most hindering in providing integrated care to patients with complex health problems.<br>• Inability to identify the right patient<br>• Current division of tasks between 1st 2nd 3rd lines<br>• Lack of capacity within general practice<br>• Absence of expertise within practice<br>• Lack of knowledge about the right approach<br>• Lack of time<br>• Insufficient priority on topic within general practice<br>• (Physical) distance between care providers<br>• The current funding of primary care<br>• An inadequate IT-system<br>• Inability to share patient data	Top 5 factors
Question 14: The list below includes factors mentioned in literature and previous research as promoting integrated care. Make a top 5 from these factors that you experience in daily practice as most conducive in providing integrated care to patients with complex health problems.<br>• Funding beyond individual condition<br>• Your motivation<br>• Motivation of others within your practice<br>• Presence of expertise within your practice<br>• Your leadership<br>• Leadership of others within your practice<br>• National (cross-domain) vision for the target group<br>• Support from the care group<br>• Existing collaborations with other caregivers<br>• Existing collaborations with social domain<br>• Existing agreements between care group and health insurer(s)	Top 5 factors
Question 15*: Would you like to elaborate on the above answers or mention an additional factor?	Yes/no
Explanation Question 15	Open question

Domain 4: Necessary developments (optional)	
The preceding questions focus on whether and why you see yourself as able or unable to provide integrated and/or person-centred care to patients with complex health problems. In closing, we would also like to look ahead to how this might be improved.	
Question 16: What can you yourself do within your practice to further promote person-centred and integrated care? Or what have you already done?	Open question
Question 17: In addition, what changes should take place within the national context of the Dutch health care system to further promote person-centred and integrated care, and who is responsible for this?	Open question

Care group questionnaire

[Consent Statement]

"This questionnaire is part of a scientific study by IQ healthcare. Please read the information below carefully and agree to participate in this study before continuing.

This questionnaire and the answers you provide will be used for scientific research purposes by IQ healthcare. Answers will be analysed and published at group level at all times. Statements or published results from this research will therefore never be traceable to you as a person or your care group. Answers will remain anonymous unless you explicitly indicate at the end of this questionnaire that we may contact you for any follow-up questions regarding your answers. Even if you indicate this, answers will still only be analysed at group level and statements or published results from this research can never be traced back to you or your care group. Answers to this questionnaire will be stored for 15 years in accordance with Dutch laws and regulations surrounding scientific research. Results of this research will be incorporated into a report that is publicly accessible and will be provided to your care group and InEen. Please provide your consent to participate below.

Please note: Without approval, this research cannot be carried out and the questionnaire will be completed immediately."

Section 1: Care group characteristics	
Question 1: What is your function within your care group?	• (programme) manager<br>• (medical) director<br>• Bundled care coordinator<br>• Framework doctor (specify area of expertise if necessary)<br>• Other (please specify below)
Question 2: How many general practices fall under your care group?	.... Practices
Question 3: In total, how many patients are currently enrolled at general practices under your care group?<br><br>Note: this includes *all* patients. Not only included in e.g., bundled payments	.... Patients

**Section 2: Current national programmes**	
In 2007, the national government decided to move towards multidisciplinary care across disciplines within primary care for patients with specific chronic conditions. In line with this new working method, a nation-wide implementation of payment bundles outside the regular registration and consultation fees was launched in 2010. This measure was initially introduced to promote cooperation between healthcare providers and concerned patients with three specific conditions (COPD, CVRM, diabetes).<br><br>The questions below mainly relate to the general policy measure of working in payment bundled for this disease-specific care. These bundles and care for these patient groups may have changed a lot within your care group. There will be some more questions about this later in this questionnaire.	
Question 4: I am familiar with the national implementation of disease-specific payment bundles	Completely agree - agree - neither agree nor disagree - disagree - completely disagree
Question 5: Within practices in our care group, following this national policy measure, disease-specific payment bundles are being used for patients with the<br>above chronic conditions (CVRM, COPD, diabetes)	Completely agree - agree - neither agree nor disagree - disagree - completely disagree
Question 6: The general concept of bundled payments for specific conditions is sufficient for providing care to patients with chronic conditions - in terms of content	Completely agree - agree - neither agree nor disagree - disagree - completely disagree
Question 7: The general concept of bundled payments for specific conditions is sufficient for providing care to patients with chronic conditions – financially speaking	Completely agree - agree - neither agree nor disagree - disagree - completely disagree
Question 8: The national implementation of the general concept of bundled payments for specific conditions encourages **integrated** care for complex patients with one or more chronic conditions	Completely agree - agree - neither agree nor disagree - disagree - completely disagree
Question 9: The national implementation of the general concept of bundled payments for specific conditions encourages **person-centred** care for complex patients with one or more chronic conditions	Completely agree - agree - neither agree nor disagree - disagree - completely disagree
In recent years, there have also been several national developments around the issue of frail elderly people. Think of:<br>• The Ministry of Health, Welfare and Sport launched the "Longer at Home Programme"<br>• A handbook on frail older people at home has been published by BeterOud	
Question 10: I am familiar with these national developments around the issue of frail elderly people	Completely agree - agree - neither agree nor disagree - disagree - completely disagree
Question 11: Within our care group, in response to these national developments, programs/initiatives have been launched specifically around the issue of frail elderly people	Completely agree - agree - neither agree nor disagree - disagree - completely disagree
Question 12: Programs/initiatives around frail elderly people within our care group have been started following the national program longer at home for frail elderly by The Ministry of Health, Welfare and Sport	Completely agree - agree - neither agree nor disagree - disagree - completely disagree
Question 13: Would you like to explain (one of your) answers to the above statements?	

**Section 3: Programmes specific to your care group - complex patients with one or more chronic**

**conditions**	
Question 14: What do you think are characterises/what would be your description of a complex patient (with or without one or more chronic conditions)?	
Question 15: Are there any initiatives within your care group that specifically address complex patients with one or more chronic conditions as described above?	[A: yes, B: no, C: don't know]
Question 16: Please provide a brief, separate description of one of the main/major initiatives that specifically focuses on complex patients with one or more chronic conditions (max three programmes in total). Please name in this description if possible:<br>• What the initiative entails<br>• When the initiative started<br>• For whom the initiative is specific<br>• What the purpose of the initiative is<br>• Which parties are involved<br>• In what way the initiative contributes to integrated care (what barriers does it remove)<br>• What barriers, if any, are/were there in the implementation (because of which initiative is perhaps not running/implemented quite optimally)	
Question 16b: The programmes listed under question 15 that specifically focus on complex patients with one or more chronic conditions encourage integrated and person-centred care for these patients.	3x [Completely agree - agree - neither agree nor disagree - disagree - completely disagree]

**Section 4: Barriers and facilitators for delivering person-centred and integrated care**	
Question 17: To what extent do you think the issues below are **barriers** for integrated and person-centred care for complex patients with one or more chronic conditions?<br>1. Inability to identify the right patient<br>2. Lack of knowledge about right approach<br>3. Funding of chronic care in disease-specific bundles<br>4. Lack of capacity within care group(s)<br>5. Absence of expertise within care group(s)<br>6. Lack of capacity within general practice<br>7. An inadequate IT system<br>8. Inability to make arrangements with health insurers<br>9. Waiting lists/insufficient availability of specialists<br>10. Inability to share patient data	
Question 18: Make a top 5 from the above factors that you consider to be most obstructive in achieving integrated and person-centred care for complex patients with one or more chronic conditions. In 1 place the factor that you consider most hindering	

Question 19: To what extent do you think the following are **promoting factors** for integrated and person-centred care for complex patients with one or more chronic conditions?<br>1. Intrinsic motivation GP<br>2. Intrinsic motivation NP<br>3. Funding beyond individual condition<br>4. Knowledge and skills of GP<br>5. Knowledge and skills of NP<br>6. Leadership of GP<br>7. Leadership of NP<br>8. National (cross-domain) vision for the target group<br>9. Support from the care group<br>10. Existing collaborations between general practice and social domain<br>11. Existing collaborations with other healthcare providers<br>12. Existing agreements between care group and health insurer(s)	
Question 20: Make a top 5 from the above factors that you consider most conducive in achieving integrated and person-centred care for complex patients with one or more chronic conditions. At 1 put the factor you consider most conducive.	
